# Supplementary material for: Factors Influencing Childhood Influenza Vaccination: A Systematic Review
Source: Vaccines (Basel). 2024 Feb 23;12(3):233. doi: 10.3390/vaccines12030233 (PMC10975121; doi:10.3390/vaccines12030233)
Supplement: Supplementary file 1 [file vaccines-12-00233-s001.zip › supplemental table S1.pdf]

Table S1. Search strategies

Medline

#	Searches	Results
1	"Influenza.mp" [Keyword] or "exp Influenza Human/"[Mesh]	96528
2	"exp Influenza Vaccines/"[Mesh] or "vaccine.mp."[Keyword] or "exp Vaccination/" [Mesh] or "vaccination.mp"[Keyword] or"exp Immunization/"[Mesh]or"immunization.mp."[Keyword]	351053
3	"exp Attitude/"or "attitude.mp."or "view.mp."or "idea.mp."or"opinion.mp. or exp Attitude/" or "exp Decision Making/ or decision.mp."or "exp Choice Behavior/ or choice.mp."or "confidence.mp."or "trust.mp."or "exp Trust/"or "acceptance.mp." or "exp Health Knowledge, Attitudes, Practice/ or exp Vaccines/ or hesitancy.mp."or "exp Awareness/ or awareness.mp."or "belief.mp."or "exp Intention/ or intention.mp."or "perception.mp. or exp Perception/"or "barrier.mp."or "facilitator.mp."or "willingness.mp."or "fear.mp. or exp Fear/"or "suspicious.mp."or "exp Vaccination Refusal or refusal.mp." or "delay.mp." or "opposition.mp."or "boycott.mp."	2847838
4	"caregiver.mp. or exp caregiver/"or "parent.mp. or exp parent/"or "exp parental attitude/ or parental.mp. or exp parental behavior/"or"children.mp. or exp child/"	2209128
5	Step 1 and Step 2 and Step 3 and Step 4	2451

Embase

#	Searches	Results
1	"influenza.mp."or"exp Influenza, Human/"	156831
2	"exp Influenza Vaccines/ or vaccine.mp." or "exp Vaccination/ or vaccination.mp."or "exp Immunization/ or immunization.mp."	553729

3	“exp Attitude/”or “attitude.mp.”or “view.mp.”or “idea.mp.”or“opinion.mp. or exp Attitude/” or “exp Decision Making/ or decision.mp.”or “exp Choice Behavior/ or choice.mp.”or “confidence.mp.”or “trust.mp.”or “exp Trust/”or “acceptance.mp.” or “exp Health Knowledge, Attitudes, Practice/ or exp Vaccines/ or hesitancy.mp.”or “exp Awareness/ or awareness.mp.”or “belief.mp.”or “exp Intention/ or intention.mp.”or “perception.mp. or exp Perception/”or “barrier.mp.”or “facilitator.mp.”or “willingness.mp.”or “fear.mp. or exp Fear/”or “suspicious.mp.”or “exp Vaccination Refusal or refusal.mp.” or “delay.mp.” or “opposition.mp.”or “boycott.mp.”	6827847
4	“caregiver.mp. or exp caregiver/”or “parent.mp. or exp parent/”or “exp parental attitude/ or parental.mp. or exp parental behavior/”or“children.mp. or exp child/”	3496737
5	Step 1 and Step 2 and Step 3 and Step 4	994

PsycINFO

#	Searches	Results
1	“influenza.mp.”or“exp Influenza, Human/”	2500
2	“exp Influenza Vaccines/ or vaccine.mp.” or “exp Vaccination/ or vaccination.mp.”or “exp Immunization/ or immunization.mp.”	7471
3	“exp Attitude/”or “attitude.mp.”or “view.mp.”or “idea.mp.”or“opinion.mp. or exp Attitude/” or “exp Decision Making/ or decision.mp.”or “exp Choice Behavior/ or choice.mp.”or “confidence.mp.”or “trust.mp.”or “exp Trust/”or “acceptance.mp.” or	1402560

	"exp Health Knowledge, Attitudes, Practice/ or exp Vaccines/ or hesitancy.mp."or "exp Awareness/ or awareness.mp."or "belief.mp."or "exp Intention/ or intention.mp."or "perception.mp. or exp Perception/"or "barrier.mp."or "facilitator.mp."or "willingness.mp."or "fear.mp. or exp Fear/"or "suspicious.mp."or "exp Vaccination Refusal or refusal.mp." or "delay.mp." or "opposition.mp."or "boycott.mp."	
4	"caregiver.mp. or exp caregiver/"or "parent.mp. or exp parent/"or "exp parental attitude/ or parental.mp. or exp parental behavior/"or"children.mp. or exp child/"	618533
5	Step 1 and Step 2 and Step 3 and Step 4	92

CINAHL Plus

#	Searches	Results
1	"Influenza* "or"flu " or MH "Influenza, Human+ " or MH "Influenza Vaccine"	29570
2	" vaccin* " or " immuniz* "or " immunis* " or MH "Immunization+"	66487
3	"attitude"or "view"or "idea"or"opinion" or "decision"or "choice"or "confidence"or "trust"or "acceptance" or "hesitancy"or "awareness"or "belief"or "intention"or "perception"or "barrier"or "facilitator"or "willingness"or "fear"or "suspicious"or "refusal" or "delay" or "opposition"or "boycott"	1130525
4	"caregiver* "or "parent* "or MH "Caregiver Burden") OR (MH "Caregiver Attitudes") or (MH "Parent-Infant Relations+") OR (MH "Parent-Child Relations+") OR (MH "Parental Attitudes+") OR (MH "Parental Behavior") OR (MH "Child+") OR "child"	752951
5	Step 1 and Step 2 and Step 3 and Step 4	817

Web of Science

#	Searches	Results
1	IELDALL FS: (Influenza)<br>DocType=All document types; Language=All languages;	103510
2	ALL=(vaccin*) OR ALL=(immuniz*)OR ALL=(immunis*)<br>DocType=All document types; Language=All languages;	416128
3	(intention) OR ALL FIELDS: (perception) OR ALL FIELDS: (barrier) OR ALL FIELDS: (facilitator) OR ALL FIELDS: (willingness) OR ALL FIELDS: (fear) OR ALL FIELDS: (suspicious) OR ALL FIELDS: (refusal) OR ALL FIELDS: (delay) OR ALL FIELDS: (opposition) OR ALL FIELDS: (boycott)<br>DocType=All document types; Language=All languages;	6647928
4	ALL=(parent*) OR ALL=(child*) OR ALL=(caregiver)<br>DocType=All document types; Language=All languages;	2862293
5	Step 1 and Step 2 and Step 3 and Step 4	1905

CNKI

#		
1	SUBJECT=流感+流感疫苗<br>Translation: SUBJECT=influenza+ influenza vaccine	/
2	SUBJECT=态度+观点+想法+意见+决定+选择+信心+信任+接受+犹豫+意识+信念+意图+观念+障碍+促进+意愿+恐惧+怀疑+拒绝+延误+反对+抵制<br>Translation:<br>SUBJECT= attitude+view+idea+opinion+decision+choice +confidence+trust+acceptance+hesitancy+awareness+belief+intention+perception+barrier+facilitator+willingness+fear+suspicious+refusal+delay+opposition+boycott”	/
3	SUBJECT=家长+父母+监护人+儿童+婴幼儿+幼儿<br>Translation:<br>SUBJECT= parent + caregiver + children + infants + young children	/
4	Step 1 and Step 2 and Step 3	449

VIP

#		
1	SUBJECT=流感+流感疫苗<br>Translation: SUBJECT=influenza+ influenza vaccine	/

2	SUBJECT=态度+观点+想法+意见+决定+选择+信心+信任+接受+犹豫+意识+信念+意图+观念+障碍+促进+意愿+恐惧+怀疑+拒绝+延误+反对+抵制<br>Translation:<br>SUBJECT= attitude+view+idea+opinion+decision+choice +confidence+trust+acceptance+hesitancy+awareness+belief+intention+perception+barrier+facilitator+willingness+fear+suspicious+refusal+delay+opposition+boycott”	/
3	SUBJECT=家长+父母+监护人+儿童+婴幼儿+幼儿<br>Translation:<br>SUBJECT= parent + caregiver + children + infants + young children	/
4	Step 1 and Step 2 and Step 3	261
