# Supplementary material for: Factors Influencing Childhood Influenza Vaccination: A Systematic Review
Source: Vaccines (Basel). 2024 Feb 23;12(3):233. doi: 10.3390/vaccines12030233 (PMC10975121; doi:10.3390/vaccines12030233)
Supplement: Supplementary file 1 [file vaccines-12-00233-s001.zip › supplemental table S4.pdf]

Supplemental file S4. Factors influencing childhood influenza vaccination

| Non-biomedical factors | Application/examples |
|---|---|
| **Knowledge** | |
| Knowledge | The participant's knowledge about the specific infection |
| Awareness | The participant's awareness of influenza as a health threat on individual or on the society as a whole |
| **Attitudes towards influenza vaccine** | |
| Attitudes towards influenza vaccination behaviors | The participant's accepting attitudes towards childhood influenza vaccination |
| Self-efficacy | The participant's perception of his/her or others' competence in engaging in vaccinating their children against influenza |
| **Perceptions of influenza and influenza vaccines** | |
| Perceived susceptibility | Self-rated health status |
| Perceived severity | The participant's assessment/ perception of the severity of the situation regarding the infection (eg, self-diagnosed symptoms experienced) |
| Perceived benefits | The participant's assessment/ perception of the benefit of receiving influenza vaccination (e.g., considering influenza vaccination to boost immunity) (misconceptions) |
| Perceived barriers | The participant's assessment/ perception of barriers to engaging in influenza vaccination (e.g., health insurance and knowledge of current policy) |
| Norm | Participants' view of how others treat illnesses |
| **Cue to action** | |
| Symptoms | Presence of fever |
| Information sources | The flow of information exchange between healthcare workers, the public and the media, etc |
| Emotion | Moods and positive or negative reactions towards vaccination or non-vaccination, including worry, anxieties, and so on |
| **Individual characteristics** | |
| Age | The age of the participant |

| | |
|---|---|
| Gender | The gender of the participant |
| Education | The education level of the participant |
| Income | The household income or monthly allowance of the participant |
| Vaccination history | Influenza vaccination behavior of family members during past influenza seasons |
| **Contextual factors** | |
| Location | The rural/urban of residence of the participant |
| Region | Region of residence of the participant —geographic area or economic development stage |
| Policy | Health policy or vaccination program that might affect access to influenza vaccine and financial incentives for recommending influenza vaccine of vaccinators |
| Access | The degree of fit between the user and the vaccination service, including accessibility, availability, acceptability, affordability, and adequacy. |
